# Maternal Chronodisruption Throughout Pregnancy Impairs Glucose Homeostasis and Adipose Tissue Physiology in the Male Rat Offspring

**DOI:** 10.3389/fendo.2021.678468

**Published:** 2021-08-16

**Authors:** Diego Halabi, Hans G. Richter, Natalia Mendez, Thilo Kähne, Carlos Spichiger, Esteban Salazar, Fabiola Torres, Karina Vergara, Maria Seron-Ferre, Claudia Torres-Farfan

**Affiliations:** ^1^Laboratory of Developmental Chronobiology, Institute of Anatomy, Histology and Pathology, Faculty of Medicine, Universidad Austral de Chile, Valdivia, Chile; ^2^Institute of Dentistry, Faculty of Medicine, Universidad Austral de Chile, Valdivia, Chile; ^3^Mass Spectrometry for Massive Proteomics, Institute of Experimental Internal Medicine, Medical Faculty, Otto von Guericke University, Magdeburg, Germany; ^4^Institute of Biochemistry and Microbiology, Faculty of Sciences, Universidad Austral de Chile, Valdivia, Chile; ^5^Programa de Fisiopatología, Instituto de Ciencias Biomédicas (ICBM), Facultad de Medicina, Universidad de Chile, Santiago, Chile

**Keywords:** DoHAD, adipose tissue, diabetes, obesity, chronobiology

## Abstract

Compelling evidence in rats support the idea that gestational chronodisruption induces major changes in maternal circadian rhythms and fetal development and that these changes impact adult life at many physiological levels. Using a model of chronic photoperiod shifting throughout gestation (CPS), in which pregnant female rats (Sprague–Dawley strain; *n* = 16 per group) were exposed to lighting schedule manipulation every 3–4 days reversing the photoperiod completely or light/dark photoperiod (12/12; LD), we explored in the adult rat male offspring body weight gain, glucose homeostasis, adipose tissue content, adipose tissue response to norepinephrine (NE), and adipose tissue proteomic in the basal condition with standard diet (SD) and in response to high-fat diet (HFD). In adult CPS male (100–200 days old; *n* = 8 per group), we found increasing body weight, under SD and adiposity. Also, we found an increased response to intraperitoneal glucose (IGTT). After 12 weeks of HFD, white adipose tissue depots in CPS offspring were increased further, and higher IGTT and lower intraperitoneal insulin tolerance response were found, despite the lack of changes in food intake. In *in vitro* experiments, we observed that adipose tissue (WAT and BAT) glycerol response to NE from CPS offspring was decreased, and it was completely abolished by HFD. At the proteomic level, in CPS adipose tissue, 275 proteins displayed differential expression, compared with LD animals fed with a standard diet. Interestingly, CPS offspring and LD fed with HFD showed 20 proteins in common (2 upregulated and 18 downregulated). Based on these common proteins, the IPA analysis found that two functional pathways were significantly altered by CPS: network 1 (AKT/ERK) and network 2 (TNF/IL4; data are available *via* ProteomeXchange with identifier PXD026315). The present data show that gestational chronodisruption induced deleterious effects in adipose tissue recruitment and function, supporting the idea that adipose tissue function was programmed *in utero* by gestational chronodisruption, inducing deficient metabolic responses that persist into adulthood.

## Introduction

At present, according to the European Foundation for the improvement of living and working conditions and the US Department of Labor, approximately 20% of the worldwide workforce is employed under shift work schedule (1, 2), increasing a risk of early onset of a series of non-communicable diseases (NCD), like metabolic syndrome, obesity, and cardiovascular diseases (1, 3–5). In this regard, compelling evidence in human and animal models support the idea that chronodisruption (i.e., disturbance of internal temporal order, essentially circadian, of endocrinology, physiology, metabolism, and behavior) might be a link between NCD and shift work schedule. Indeed, similar phenomena arose during pregnancy, namely, gestational chronodisruption (6–9). In human, exposure to shift work schedule during pregnancy has been associated with an increased risk of miscarriage, preterm delivery, and low birth weight, in addition to higher incidence of sleep and metabolic and cardiovascular disturbances in the offspring (10–12). Unfortunately, interpreting human studies during pregnancy is quite difficult, due to confusing scenarios imposed by the effects of food availability, electronic screen exposure, emotional support, and family life. Actually, new epidemiological studies add more evidence about the potential deleterious effects of gestational chronodisruption in human, although conducting studies with more controlled conditions is imperative (5, 13, 14).

Therefore, to dissect the potential mechanism involved in the long-term effect of chronodisruption, we use a model in which pregnant female rats were exposed to lighting schedule manipulation every 3–4 days reversing completely their photoperiod (chronic photoperiod shift, CPS), simulating night shift work schedules in humans (6, 9). *In vivo* and *in vitro* experiments in rat, non-human primates, and sheep demonstrated that gestational chronodisruption affects fetal organ function like adrenal, heart, hippocampus, liver, and fetal hormonal rhythms like prolactin, corticosterone, and cortisol. Altogether, the current evidence supports the idea that gestational chronodisruption is indeed an unhealthy signal for fetal development. Moreover, adult offspring of these studies show changes in organs such as liver, kidneys, and pancreas and an increase in the amount of adipose tissue (6–8, 15–20). Interestingly, in precocious species like sheep, new information support the notion that the timing at which the effect of chronodisruption appears could be related to age. In a recent study, Gatford et al. (21) found a lack of or a weak effect of maternal chronodisruption in young sheep, in contrast to that reported previously in young rats (9).

Adipose tissue has been proposed as an important target of developmental programming since obesity is the main risk factor for numerous pathologies, such as type 2 diabetes mellitus, insulin resistance (22), hypertension (23), cardiometabolic disease (24), and some types of cancer (25). Obesity is observed clinically by an excessive accumulation of white adipose tissue, related to a state of chronic and mild inflammation, which is directly related to the complications generated by obesity (26). It must be kept in mind that in mammals, there are two types of adipose tissue that are structurally and functionally different: white adipose and brown adipose tissue. White adipose tissue (WAT) is responsible for energy storage in the form of triglycerides, and it plays an endocrine function through the secretion of inflammatory cytokines. Meanwhile, brown adipose tissue (BAT) is responsible for energy dissipation as heat, which is produced from triglycerides stored in brown adipocytes, a physiological process called thermogenesis (27, 28).

Using the rat model of CPS throughout gestation, we investigated the long-term detrimental effects of gestational chronodisruption on glucose homeostasis and adipose tissue physiology and metabolism in the male adult offspring, based on our previous studies in rat male offspring in which we documented the long-term effect of the cardiovascular, endocrine, and metabolic impact of gestational chronodisruption in the offspring (6). In this model, as in other models of developmental origin of health and diseases (29), important sex differences have been shown in the offspring (9). Furthermore, considering risk factors prevailing in a modern society subjected to night shift work schedules, the impact of a second cardiometabolic challenge [high-fat diet (HFD) for 12 weeks] was evaluated in the male adult offspring that had been gestated under CPS relative to LD (control) conditions. An integrative array of methodologies was applied to generate the following outcomes: food intake and cumulative weight gain, glucose and insulin tolerance, fasting glucose and insulin levels, serum leptin and adiponectin levels, WAT and BAT tissue and cellular characterization, *in vitro* WAT and BAT glycerol response to norepinephrine, global genomic DNA methylation, and quantitative proteomics analyses.

## Materials And Methods

### Animals

The protocols were approved by the Bioethics Commission from the Universidad Austral de Chile (CBA: 352/2019). Animal handling was performed following the guidelines for the care and use of laboratory animals of the Institute for Laboratory Animals Research of the National Research Council.

We raised and maintained 32 female rats of Sprague-Dawley strain obtained from Charles River (CRL International Inc., Kingston NY). These rats were mated, and the pregnancy was determined by the presence of positive sperm, obtained by vaginal smears, calling that day “day 0 of gestation” (E0). All pregnant rats had water and standard food (Prolab^®^ RMH 3000, Lab diet, USA) *ad libitum* under controlled temperature (20°C–22°C) in standard cages inside a cabinet with filters and ventilation. From day 1 of gestation, pregnant rats were randomly separated into two groups of light/dark photoperiod:

A) Control light/dark (LD *n* = 16 pregnant female): From day 1 of gestation, a group of dams continued with the light/dark 12:12 photoperiod, with artificial white light ~400 lux at the head level, which is turned on at 0700 h and turned off at 1900 h.

B) Chronic phase shift of photoperiod (CPS *n* = 16 pregnant females): using a similar protocol reported by us (6). Briefly, pregnant females were exposed to lighting schedule manipulation every 3–4 days reversing completely the photoperiod. The photoperiod reversal occurred at the night of day 0 of gestation, so that lights, rather than going off at 1900 h, remained on until 0700 h of day 2. At 18 days of gestation, the mothers returned to a normal 24-h photoperiod (12:12, lights on at 0700 h) and continued in this photoperiod thereafter. Exposure of pregnant females to CPS had no impact on food consumption and maternal weight (6).

Exposure of pregnant females to CPS had no impact on daily food consumption (monitored every week) through pregnancy (LD: 21.2 ± 0.6 g/day, *n* = 16 *vs.* CPS: 22.3 ± 0.6, *n* = 16), maternal weight at the end of gestation (LD: 427.6 ± 6.8 g, *n* = 16 *vs.* CPS: 430.3 ± 10.6, *n* = 16), or maternal weight gain at the end of gestation (LD: 134.4 ± 2.8 g, *n* = 16 *vs.* CPS: 135.0 ± 7.5 g, *n* = 16) as reported previously (6).

**Figure 1:**
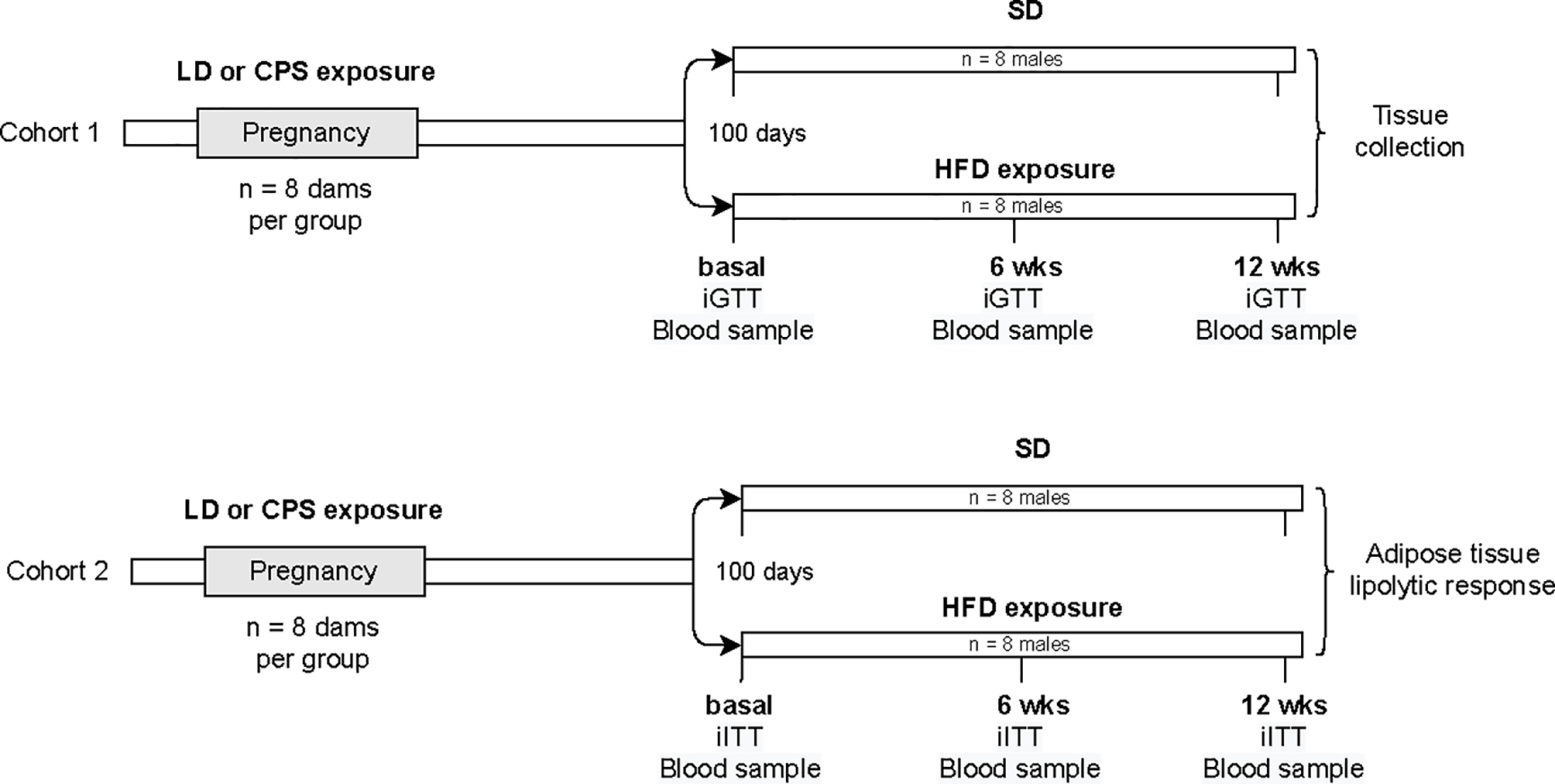
Flow diagram of experimental protocol. Pregnant rats were exposed to either control photoperiod (LD) or chronic photoperiod shift (CPS) from mating until 18 days of gestation. At 100 days old, male offspring were exposed to either high-fat diet (HFD; 45% excess calories) or standard diet (SD) for 12 weeks (about 200 days old). iGTT, intraperitoneal glucose tolerance test; iITT, intraperitoneal insulin tolerance test; *n* = number of animals.

At birth, mothers and their pups were maintained in control photoperiod 12:12, and the male offspring were studied. All the animals were housed individually after weaning and weighted weekly, and food consumption was measured by weighing the food placed in the cage and the amount left 3 days later, when bed and fresh food were replaced. For the current study, we generated two cohorts of animals separated and used them in the protocols described below **(** **Figure 1**). At 100 days of age, two brothers from the same pre-natal condition were separated and one brother was exposed to a HFD (45% excess calories) for 12 weeks and paired with a brother fed with standard food (Prolab^®^ RMH 3000, Lab diet, USA). Intraperitoneal glucose tolerance test (iGTT, Cohort1) and intraperitoneal insulin tolerance test (iITT, Cohort 2) were performed at 100 days (basal) and 12 weeks after exposure to either a HFD or standard diet. In addition, only in those males exposed to HFD was an additional iGTT and iITT test performed after 6 weeks under HFD. One week after the last test, animals were euthanized and adipose tissue was collected.

### Intraperitoneal Glucose Tolerance Test

Male rats (*n* = 8 per group-Cohort 1) at 0800 h (after 12-h fasting) were anesthetized (Isoflurane 2.5%–3.5%) and injected with intraperitoneal glucose (1 g/kg; Glucose, Sanderson laboratories; Chile). A blood drop was collected from the tail to measure glucose levels (Accu-Chek; Roche Diagnostics) at −15 and 0 min before glucose administration and 30, 60, 90, 120, and 180 min after glucose injection. A blood sample was collected at −15 min to measure fasting glucose, insulin, leptin and adiponectin.

### Intraperitoneal Insulin Tolerance Test

Male rats (*n* = 8 per group-Cohort 2) at 1500 h were anesthetized and injected with insulin (1U/kg; Humalog^®^ #CAT: VL7510, Eli Lilly and Company, Indianapolis, USA) after 6-h fasting. A blood drop was collected from the tail to measure glucose levels (Accu-Chek; Roche Diagnostics) at 0, 15, 25, 35, 45, and 60 min after glucose injection. A blood sample was collected at 0 min to measure fasting glucose, insulin, leptin, and adiponectin.

### Adipose Tissue Depots

At 200 days of age (after 12 weeks of HDF, *n* = 8 per group—Cohort 1), eight male rats per group were deeply anesthetized (Isoflurane 3%, Baxter Laboratories, Melbourne, Australia), a midline incision was done to expose the *vena cava*, and an overdose of sodium thiopental (150 mg/kg; Vetpharma, Buenos Aires, Argentina) was administered. Immediately after death was confirmed, whole white adipose depots (inguinal, epigonadal, and perirenal) and interscapular brown adipose depot were dissected and weighted. Pieces were stored at −80°C for proteomic analysis or fixed in 4% formalin for histological analysis. The remaining adipose tissue were stored in our tissue bank at −80°C.

### Adipose Tissue Lipolytic Response to Norepinephrine *In Vitro*

(*n* = 8 per group—Cohort 2). Epigonadal white adipose tissue (eWAT) and interscapular brown adipose tissue (iBAT) were dissected from individual rats, cut in small explants (about 50 mg for eWAT and 25 mg for iBAT) and suspended in culture medium (D-MEM F12, Sigma-Aldrich, St. Louis, MO, USA). Explants (10 explants for each animal) were pre-incubated in culture medium for 6 h at 37°C and aerated with 95% air CO_2_ and 5% O_2_. Next, explants were incubated in duplicate for 6 h in 2 ml medium alone (basal) or containing 0.01, 0.1, 1, and 10 μM of norepinephrine [A7257 (−)-Norepinephrine, Sigma-Aldrich, St. Louis, MO, USA]. At the end of incubation, the supernatant was collected, total protein of explants was assayed by Bradford method, and lipolysis was determined measuring the glycerol present in the supernatant fraction with a commercial kit (FG0100 Free Glycerol Determination Kit, Sigma-Aldrich, St. Louis, MO, USA). Production of glycerol was calculated as microgram per milligram of total protein in the explant.

### Hormone Assays

Blood samples were collected from the tail of male rats (*n* = 8 per group—Cohort 1) after 12-h fasting (*n* = 8 per group) at basal, 6 weeks, and 12 weeks after HFD exposure. The serum of each sample was obtained by centrifugation (4,000×*g*, 10 min, 22°C), and insulin, leptin, and adiponectin concentrations were measured by a commercial immunoassay kit according to the manufacturer’s instructions (MILLIPLEX^®^ MAP Kit; Merck, KGaA, Darmstadt, Germany). The inter- and intra-assay coefficients were less than 10%.

### Global Methylation

Adipose tissue (prWAT and iBAT, *n* = 5 CPS + SD and LD + SD; 90 days old), stored in our tissue bank from previous studies (7, 30), were used to evaluate global genomic DNA methylation using MethylFlash (methylated DNA quantification colorimetric kit; Epigentek Group Inc., Farmingdale, NY, USA), following the manufacturer’s instructions. Basically, our aim was to compare the effect of gestational chronodisruption in global methylation in adipose tissue since epigenetic changes have been reported previously by us in adrenal and kidney, organs in which such changes have been accompanied with changes in organ function already in fetal life.

### Proteomic Analyses

Protein identification was performed by high-resolution mass spectrometry on a hybrid dual-pressure linear ion trap/orbitrap mass spectrometer (LTQ Orbitrap Velos Pro, Thermo Scientific, San Jose, CA, USA) equipped with an EASY-nLC Ultra HPLC (Thermo Scientific). Input was 50 mg of white adipose tissue for each sample (*n* = 8 per group). For analysis, peptide samples were adjusted to 10 μl 1% ACN/0.1% TFA and fractionated on a 75-μm (ID), 25-cm PepMap C18-column, packed with 2 μm resin (Dionex/Thermo Scientific). The separation was achieved through applying a gradient from 2% to 35% ACN in 0.1% formic acid over 150 min at a flow rate of 300 nl/min. An Orbitrap full MS scan was followed by up to 20 LTQ MS/MS runs using collision-induced dissociation (CID) fragmentation of the most abundantly detected peptide ions. Essential MS settings were as follows: full MS (FTMS; resolution 60,000; m/z range 400–2000); MS/MS (Linear Trap; minimum signal threshold 500; isolation width 2 Da; dynamic exclusion time setting 30 s; singly charged ions were excluded from selection). The normalized collision energy was set to 35%, and activation time was set to 10 ms. The mass spectrometry proteomics data have been deposited to the ProteomeXchange Consortium *via* the PRIDE (31) partner repository with the dataset identifier PXD026315.

### Statistical Analysis

Data are expressed as mean ± standard error of the mean (SEM). LD and CPS differences in weight gain, food consumption, glycerol release, hormone levels, iGTT, iITT, and global methylation were analyzed by two-way repeated measures ANOVA and Bonferroni post-hoc test when group factor or interaction factor was significant (32). Additionally, we calculated the area under the curve (AUC) for iGTT and area above the curve (AAC) for iITT. Statistical analyses were performed using GraphPad Prism (version 9; GraphPad Software Inc., San Diego, CA). Results were considered significant when *p* < 0.05. For adipose tissue proteomics analysis, raw data processing and protein identification were performed using PEAKS Studio V.8.0 (Bioinformatics Solutions, Waterloo, Canada). False discovery rate was set to <1%. Label-free quantification was performed using Progenesis QI for proteomics (Nonlinear Dynamics/Waters). Proteins with abundance ratios of >1.5 or <1/1.5 at *p* < 0.05 were considered as significantly regulated. We applied an integrative and unbiased analysis approach for functional analysis of the proteins with significant changes in expression against the control group (LD offspring). Ingenuity Pathways Analysis (IPA) computes a score for each protein network according to the fit of that network to the user-defined set of “focus protein.” The score is derived from a *p*-value and indicates the likelihood of the focus proteins in a network being found together due to random chance. A score of 2 indicates that there is a 1 in 100 chance that the focus proteins are together in a network due to random chance. Therefore, scores of 2 or higher have at least a 99% confidence of not being generated by random chance alone [for details, see (17)]. First, we identified the modified proteins for the LD + HFD, CPS and CPS + HFD conditions compared to LD (fold change > 1.5; *p* < 0.05). After this exploratory analysis, we decided to focus on the common proteins for the CPS and LD + HFD groups, based on our observed results. Under this criterion, we selected the 20 proteins shown in **Figure 8** and were grouped to build a new dataset and analyzed by IPA to generate the interactomes.

## Results

### Effects of Gestational CPS on the Offspring’s Metabolic Status

**Figure 2:**
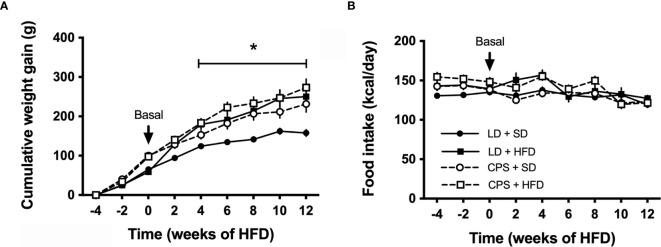
Effects of gestational chronic phase shift of photoperiod on **(A)** cumulative weight gain and **(B)** food intake of rats fed with standard diet (SD) and 45% high-fat diet (HFD) for 12 weeks. Data are mean ± SEM. Solid line (LD): adult offspring gestated under control photoperiod; dashed line (CPS): adult offspring that had been gestated under chronic photoperiod shift; circles: adult offspring fed with standard diet Prolab^®^ RMH 3000; squares: adult offspring fed with HFD. *n* = 8 per group. *LD + HFD; CPS + SD and CPS + HFD are different from LD + SD (*p* < 0.05 pairwise comparison at each time point with Bonferroni correction).

Between weaning (21 days old) and 100 days of age, CPS and LD animals had similar food consumption expressed as kcal/day (LD: 137 ± 12 kcal/day, *n* = 16 *versus* CPS: 143 ± 14, *n* = 16; *p* = 0.197 unpaired *t*-test). However, CPS offspring was slightly heavier (LD: 602 ± 51 g, *n* = 16 *versus* CPS: 615 ± 50 g, *n* = 16; *p* = 0.5, unpaired *t*-test). As shown in **Figure 2A**, CPS animals fed a standard diet (CPS + SD) between 100 and 200 days became increasingly heavier than LD + ST animals. After the 4 weeks under HFD, LD + HFD and CPS + HFD animals showed a significant increase in body weight *versus* LD + SD (time × group factor *p* < 0.001; **Figure 2A**). Thus, CPS animals with SD or HFD featured a similar weight gain profile, reaching an increase of about 50% *versus* LD + SD (**Figure 2A**). Food intake remains steady along the 12 weeks in animals receiving either standard or HFD (**Figure 2B**). Therefore, CPS animals fed with SD had a similar weight gain to those LD or CPS fed with HFD.

**Figure 3:**
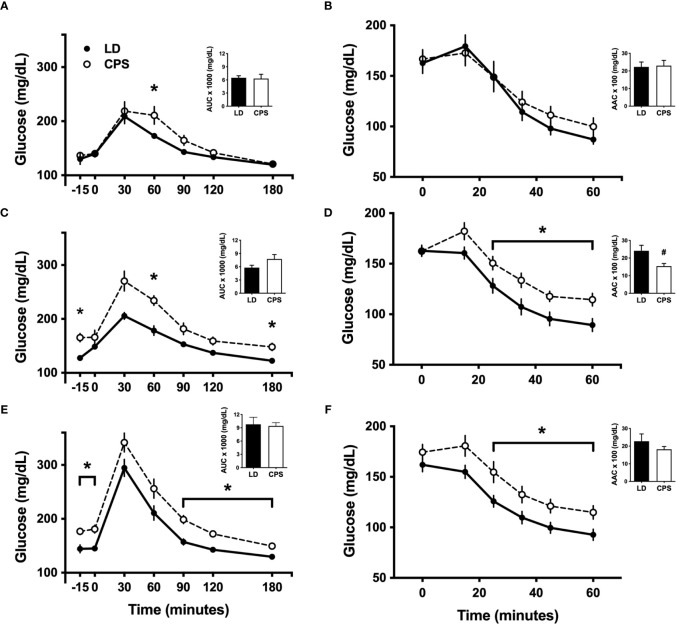
Effects of gestational chronic phase shift of photoperiod on intraperitoneal glucose tolerance test **(A, C, E)** and intraperitoneal insulin tolerance test **(B, D, F)** of rats fed with 45% high-fat diet (HFD) for 12 weeks, measured at week 0/basal **(A, B)**, 6 weeks **(C, D)** and 12 weeks **(E, F)**. Data are mean ± SEM. Solid line: LD, adult rats that had been gestated under control 12:12 (LD) photoperiod; dashed line: CPS, adult rats that had been gestated under chronic phase shift (CPS) photoperiod. AUC, Area under the curve; AAC, Area above the curve. *n* = 8 per group. *different from LD (*p* < 0.05 pairwise comparison at each time point with Bonferroni correction), ^#^different from LD (*p* < 0.05, unpaired *t*-test).

**Figure 4:**
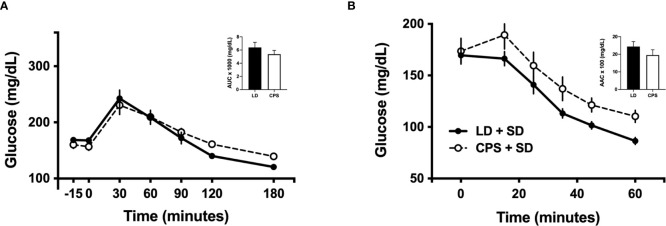
Effects of gestational chronic phase shift of photoperiod on intraperitoneal glucose tolerance test **(A)** and intraperitoneal insulin tolerance test **(B)** of rats fed with standard diet (SD) since 100 to 200 days of age. Data are mean ± SEM. Solid line: LD, adult rats that had been gestated under control 12:12 (LD) photoperiod; dashed line: CPS, adult rats that had been gestated under chronic phase shift (CPS) photoperiod; circles: adult offspring fed with standard diet Prolab^®^ RMH 3000; squares: adult offspring fed with HFD. *n* = 8 per group.

In line with a previous report (6), we found slight differences in intraperitoneal glucose tolerance test (iGTT) between LD and CPS adult offspring at 100 days of age, before the beginning of HFD (**Figure 3A**). After 60 min post glucose injection, glucose levels rapidly decreased to 173± 2 mg/dl in the LD group, remaining elevated in the CPS group (210 ± 16 mg/dl; *p* = 0.024; two-way ANOVA and Bonferroni test), but no interaction time × column was found (*p* = 0.172). In addition, analyzed as AUC, glucose response displayed a similar value to the CPS group without reaching statistical significance (*p* = 0.857, unpaired *t*-test; **Figure 3A**). However, no difference was found at 200 days (**Figure 4A**). Important differences were found in iGTT after 6 weeks with HFD. At this time window, the adult offspring gestated under LD conditions maintained the glucose response similar to that observed at week 0. In contrast, a marked increase in glucose response was observed in the CPS group relative to control LD (time × group factor: *p* = 0.012, two-way ANOVA and Bonferroni; **Figure 3C**). Analyzed as AUC, glucose response remained the same as week 0 (basal) for the LD group, while basal plasma glucose levels tended to increase in the CPS group relative to control LD group (*p* = 0.111; unpaired *t*-test). As anticipated, a significant increase in glucose response was found in LD animals after 12 weeks (**Figure 3C**) regarding 0 and 6 weeks of HFD. At this late time window, the adult CPS offspring presented significantly higher levels of glucose in basal conditions, raising up to 340 mg/dl levels after 30 min post intraperitoneal glucose injection (**Figure 3E**), but no interaction time × group was found (*p* = 0.622). Measured as AUC, the glucose response was similar to the CPS group compared to the LD group (*p* = 0.838; unpaired *t*-test), probably due to the fact that the CPS group begins with higher basal plasma levels.

Intraperitoneal insulin tolerance tests were carried out in the parallel cohort 2. One-hundred-day-old animals have shown no difference between LD and CPS offspring (time × group factor: *p* = 0.616, two-way ANOVA; **Figure 3B**), expressed either as plasma glucose levels or area above the curve (AAC; *p* = 0.866 unpaired *t*-test). Similar results were found at 200 days of age (**Figure 4B**). After 6 weeks of HFD, the group that had been gestated under LD presented a glucose response to insulin alike to the one observed at week 0 (basal). However, the CPS group displayed a marked increase relative to both CPS and LD at week 0 (basal) and to LD at week 6 (time × group factor: *p* = 0.026, two-way ANOVA and Bonferroni; **Figure 3D**). Glucose response expressed as AAC was at the same level in the LD group regarding week 0 (basal), while CPS showed a significant decrease of AAC (*p* = 0.033; unpaired *t*-test). Finally, a third iITT was performed after 12 weeks under HFD (**Figure 3F**). No significant increase in glucose response to insulin was found in LD animals regarding week 0 (basal). Meanwhile, CPS animals presented significantly higher levels of glucose in basal conditions, maintaining higher levels of glucose up to the end of the experiment (time × group factor: *p* = 0.019, two-way ANOVA and Bonferroni). However, AAC values for insulin response were lower in the CPS compared to the LD group, but not statistically significant (*p* = 0.325, unpaired *t*-test; **Figure 3F**), probably due to the higher fasting glucose in CPS.

**Figure 5:**
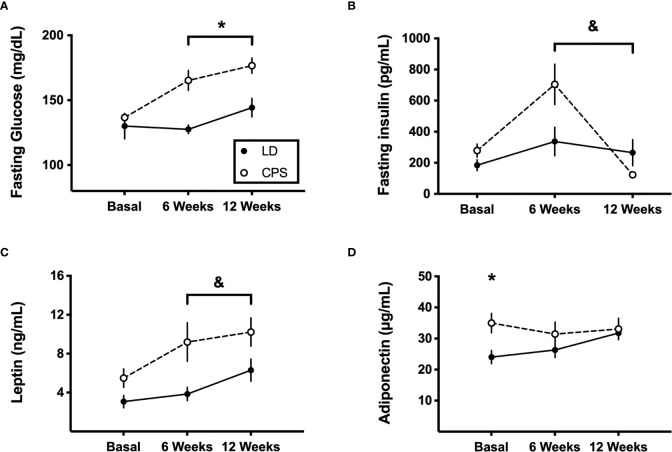
Effects of gestational chronic phase shift of photoperiod on serum levels of fasting glucose **(A)**, fasting insulin **(B)**, leptin **(C)**, and adiponectin **(D)** at 6 and 12 weeks of 45% high-fat diet intake. Data are mean ± SEM. Solid line: LD, adult rats that had been gestated under control 12:12 (LD) photoperiod. Dashed line: CPS, adult rats that had been gestated under chronic phase shift (CPS) photoperiod. *n* = 8 per group. *Different from LD (*p* < 0.05; two-way ANOVA and Bonferroni test). ^&^Different from basal (*p* < 0.01; two-way ANOVA and Bonferroni test).

Next, we evaluated the potential role of the endocrine system to help to explain our findings. To this goal, insulin, leptin, and adiponectin plasma levels were measured before applying the glucose tolerance test in fasting conditions after HFD. First, steady levels of fasting glucose and circulating insulin, leptin, and adiponectin were found throughout 12 weeks under HFD challenge in the control LD group (**Figure 5**). In contrast, adult CPS offspring displayed increased levels of fasting glucose (time × group factor: *p* = 0.025, two-way ANOVA and Bonferroni; **Figure 5A**) at 6 and 12 weeks of treatment. In addition, they exhibited high insulin plasma levels at 6 weeks (time × group factor: *p* = 0.016, two-way ANOVA and Bonferroni; **Figure 5B**, i e, week 6 versus week 0/basal), dropping to minimal levels after 12 weeks of HFD challenge (i.e., week 12 *versus* week 6). On the other hand, when plasma levels of the two hormones produced by WAT from CPS animals were evaluated, leptin showed constantly increased levels along the 12 weeks under HFD, which is consistent with higher body weight gain (**Figure 5C**), without an interaction (time × group factor: *p* = 0.123, two-way ANOVA). Adiponectin displayed higher levels already in basal conditions in CPS offspring (i.e., before HFD), but like the levels measured in the control LD offspring, with stable values throughout HFD challenge (time × group factor: *p* = 0.149, two-way ANOVA; **Figure 5D**).

### Effects of Gestational CPS on the Offspring’s Adipose Tissue Lipolytic Function *In Vitro*

**Figure 6:**
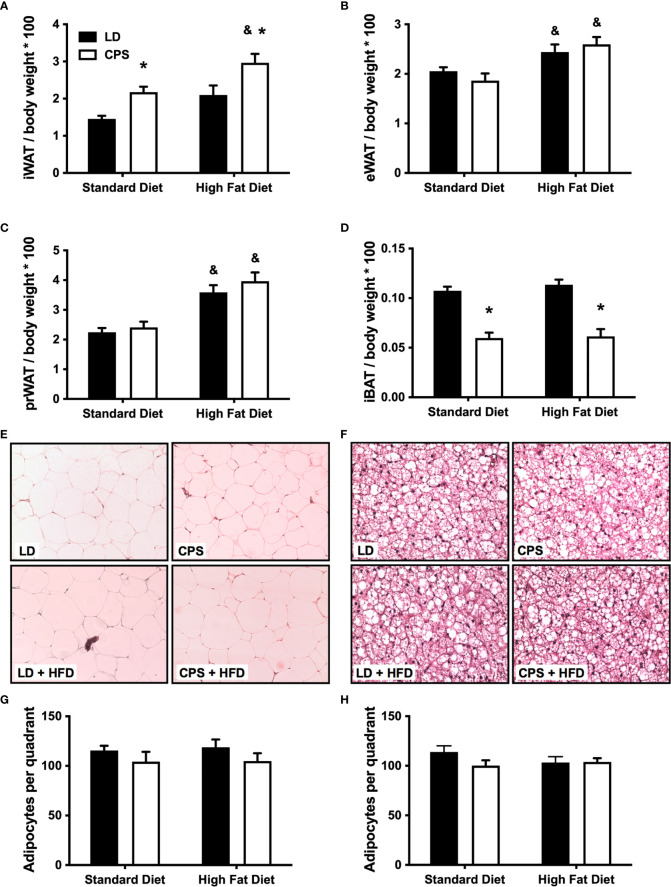
Effects of gestational chronic phase shift of photoperiod in recruitment of adipose tissue depot. Upper panel displays the weight of whole adipose tissue depots adjusted by full-body weight of 200-day-old rats fed with standard diet or high-fat diet. **(A)** Inguinal adipose tissue, **(B)** epigonadal adipose tissue, **(C)** perirenal adipose tissue, **(D)** interscapular brown adipose tissue. Middle panel displays representative H&E staining of **(E)** 10× perirenal white adipose tissue and **(F)** 40× interscapular adipose tissue histological sections. Lower panel displays the corresponding cell number counts as shown in **(G)** from white adipose tissue and **(H)** from brown adipose tissue. Data are mean ± SEM. Black bars: LD, adult rats that had been gestated under control 12:12 (LD) photoperiod. White bars: CPS, adult rats that had been gestated under chronic phase shift (CPS) photoperiod. Standard diet: animals fed with Prolab^®^ RMH 3000, High-fat diet: animals fed with 45% high-fat diet for 12 weeks. *n* = 8 per group. *LD + HFD; CPS + SD and CPS + HFD are different from LD + SD (*p* < 0.05 pairwise comparison at each time point with Bonferroni correction). ^&^Different from standard diet (*p* < 0.05 pairwise comparison at each time point with Bonferroni correction).

White and brown adipose tissues were collected and analyzed 1 week after the last test described above was carried out. CPS animals fed standard diet had more iWAT and less BAT than animals gestated under control (LD) conditions, while pWAT and eWAT show no difference. HFD induced an increase in perirenal, epigonadal, and inguinal white adipose tissue pads (**Figures 6A–C**), matching findings from another report (33). Meanwhile, CPS offspring displayed increased subcutaneous iWAT depot with both standard diet and HFD, compared to LD without interaction (column factor: <0.001; time × group factor: *p* = 0.704, two-way ANOVA). Surprisingly, CPS iWAT levels under standard diet were similar to those of LD + HFD iWAT. We explored whether the treatments induced an increase in the number of white adipose cells in eWAT. Neither LD nor CPS with or without HFD was associated with differences in the number of cells per section, supporting the idea that the effects induced by HFD or CPS on eWAT total mass represent bigger fat pads. Next, we evaluated the effects of CPS and HFD on brown adipose tissue depot. As expected, HFD given to the control LD offspring did not affect the content of brown adipose tissue (interscapular depot). However, CPS offspring presented a significant decrease (about 50%; group factor: *p* < 0.001, two-way ANOVA and Bonferroni) in iBAT, which was not affected by HFD (time × group factor: *p* = 0.697, two-way ANOVA; (**Figure 6D**). Neither LD nor CPS with or without HFD displayed differences in the number of cells per section (**Figures 6E–H**).

**Figure 7:**
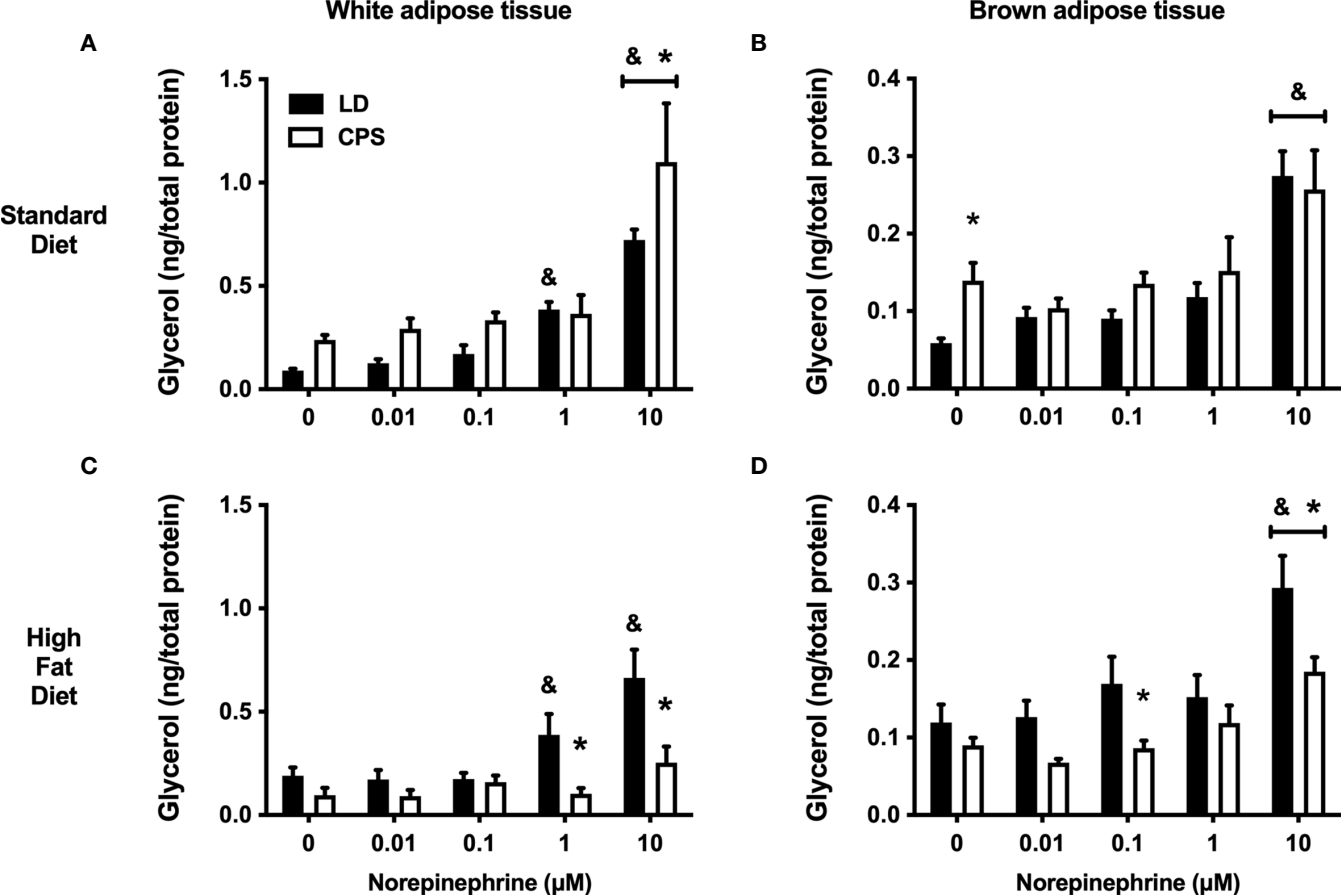
Effects of gestational chronic phase shift of photoperiod on glycerol response to increasing doses of norepinephrine (NE) of white epigonadal adipose tissue **(A, C)** and brown interscapular adipose tissue **(B, D)**. Explants from 200-day-old rats fed with either standard diet Prolab^®^ RMH 3000 **(A, B)** or 45% high-fat diet (HFD; C, D). Data are mean ± SEM. Black bars: LD, adult rats that had been gestated under control 12:12 (LD) photoperiod. White bars: CPS, adult rats that had been gestated under chronic phase shift (CPS) photoperiod. *n* = 8 per group. *different from LD. ^&^Different from basal LD (0 μM NE). All comparisons: *p* < 0.05 pairwise comparison at each time point with Bonferroni correction.

The functional integrity of WAT was assessed under *in vitro* conditions by testing glycerol response to increasing doses of norepinephrine (NE). Explants of LD eWAT animals fed with standard diet had a significant glycerol response to 1 µM NE and reached a maximal response with 10 µM NE. In contrast, in CPS, eWAT explants’ glycerol response to NE was statistically significant only to 10 µM NE (group factor: *p* = 0.103, two-way ANOVA and Bonferroni; **Figure 7A**). In animals fed with HFD, the glycerol response to NE in eWAT LD explants was similar to the one found under the standard diet (group factor: *p* = 0.64, treatment × group factor: *p* = 0.76, two-way ANOVA and Bonferroni; **Figures 7A–C**). Therefore, HFD did not affect the *in vitro* response to NE in LD conditions (**Figures 7C**). In contrast, eWAT explants from adult animals that had been gestated under CPS conditions displayed a blunted glycerol response to all NE doses tested (group factor: *p* < 0.001, treatment × group factor: *p* = 0.024, two-way ANOVA and Bonferroni; **Figure 7C**).

The functionality of BAT was also evaluated under *in vitro* conditions. First, BAT explants of adult offspring gestated under LD and CPS conditions and fed with standard diet showed increased glycerol response to 10 µM NE (group factor: *p* = 0.029, two-way ANOVA and Bonferroni; **Figure 7B**). Of note, higher basal levels of glycerol were found in the incubation media from CPS animals than that of control (LD) animals. This difference was no longer present in BAT explants from CPS animals receiving HFD for 12 weeks. In addition, BAT explants from these animals showed a reduced glycerol response to NE and overall glycerol production was lower than that of BAT explants from control (LD) conditions also fed on HFD (group factor: *p* < 0.001, treatment × group factor: *p* = 0.440, two-way ANOVA and Bonferroni; **Figure 7D**).

**Figure 8:**
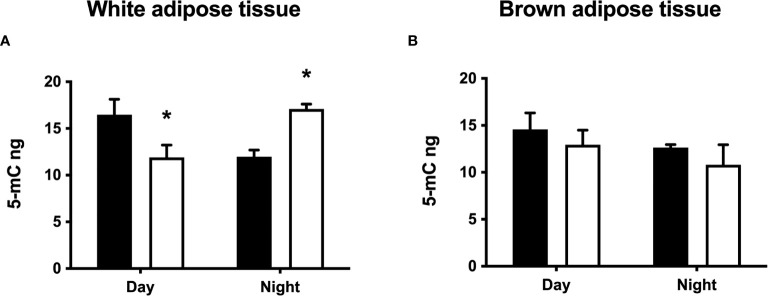
Effects of gestational chronic phase shift of photoperiod on global genomic DNA methylation of perirenal white adipose tissue **(A)** and interscapular brown adipose tissue **(B)** from 90-day-old rats sampled at day (10:00 h) and night (22:00 h). Black bars: LD, adult rats that had been gestated under control 12:12 (LD) photoperiod. White bars: CPS, adult rats that had been gestated under chronic phase shift (CPS) photoperiod. *n* = 5 per group. *different from LD (*p* < 0.05 pairwise comparison at each time point with Bonferroni correction).

Some of the differences in white and brown adipose tissue between CPS and LD animals described above were present before the HFD challenge. Therefore, we explored potential epigenetic mechanisms involved by measuring global genomic DNA methylation status in both WAT and BAT depots in the offspring at 100 days. Perirenal WAT from LD offspring showed significant day/night methylation differences, with levels that were about 25% higher during daytime (**Figure 8A**). Conversely, lower levels of global genomic DNA methylation were detected during daytime in perirenal WAT from CPS offspring (time × group factor: *p* = 0.001, two-way ANOVA and Bonferroni). It should be noted that the mean day/night methylation level was similar for perirenal WAT from both LD and CPS adult offspring. Finally, no differences were found for global DNA methylation in BAT, either during daytime or nighttime (*p* = 0.948, two-way ANOVA; **Figure 8B**). These early differences in global DNA methylation suggest that epigenetic programming has occurred in WAT from the CPS group.

Next, we evaluated the effects of prenatal chronodisruption and postnatal HFD on total protein expression in white adipose tissue using a quantitative proteomics analysis. (Data are available *via* ProteomeXchange with identifier PXD026315.)

Animals that had been gestated under LD conditions and received HFD during adult life displayed a significant increase in body weight and weight of white adipose tissue fat pads at 100 days. These exacerbated weight-gain pattern induced by HFD was accompanied by modification in the expression level of 91 proteins (fold change higher than 1.5) in white adipose tissue. We further analyzed the proteomics data using Ingenuity Pathway Analysis (IPA) to look for interactions among the differentially expressed set of proteins framed in functional pathways. As might be expected, the major effects at the proteome level were found in the functional pathway involving fatty acid metabolism. In addition, changes in the expression level of proteins related to cytoskeleton function and cellular differentiation were found.

WAT from adult CPS offspring at 200 days fed with SD displayed differential expression of 275 proteins with a fold change higher than 1.5, when compared to the control LD group fed with SD. Remarkably, 96% of the differentially expressed proteins were downregulated in WAT from adult CPS offspring. This significant proportion of downregulated proteins is consistent with the metabolic alterations described above for WAT from adult CPS relative to LD offspring.

Comparison of adult CPS offspring fed standard *versus* CPS fed HFD led to the identification of 235 proteins exhibiting significantly modified expression levels, that is to say, 114 more proteins than LD animals receiving HFD. Among these, 219 proteins were upregulated and 16 proteins were downregulated.

**Figure 9:**
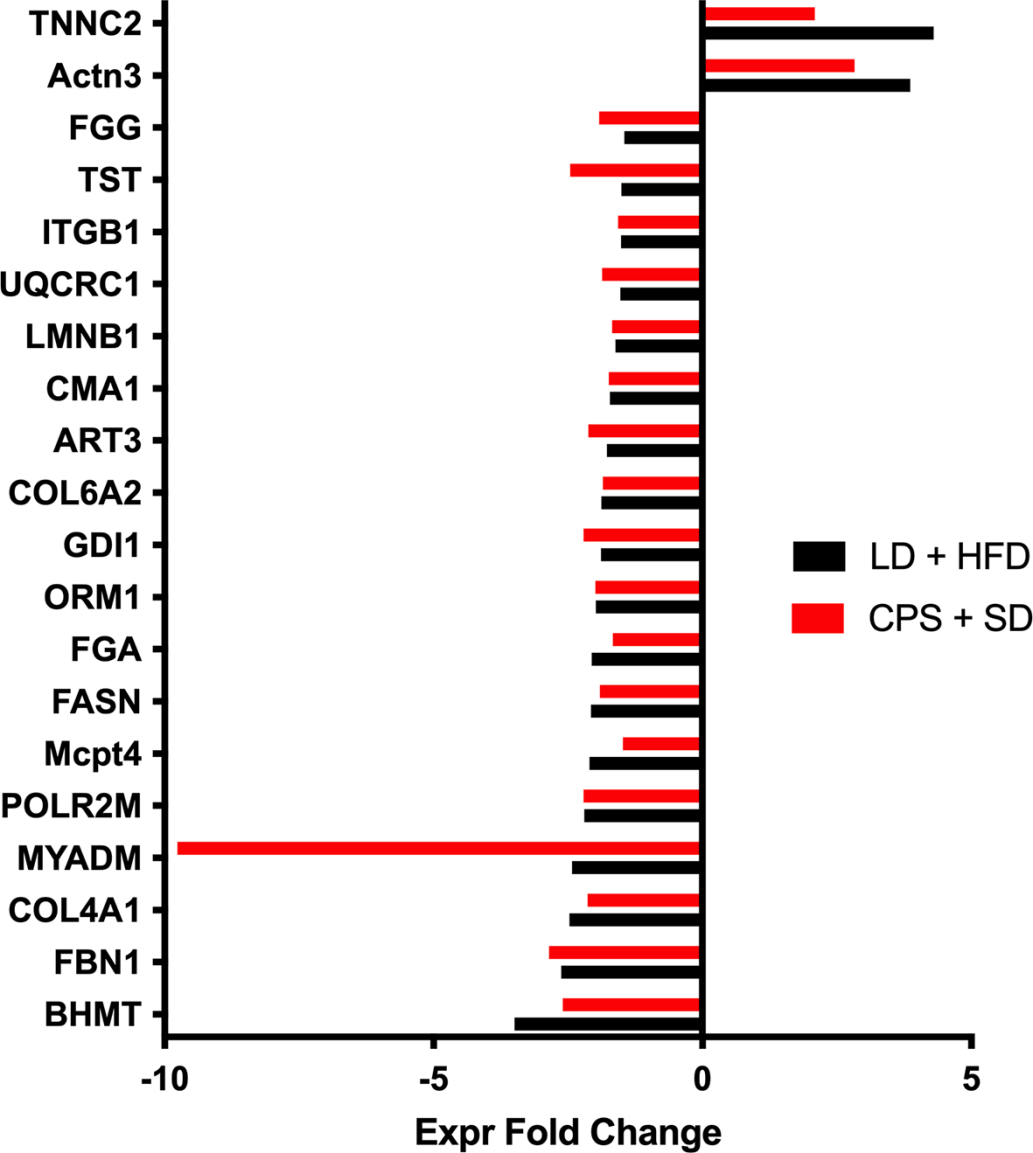
Up and down expressed proteins in WAT from adult offspring gestated under control conditions and fed with high-fat diet during 12 weeks from postnatal day 100 onwards (LD + HFD group—black bar) and adult offspring gestated under CPS conditions and fed with standard diet postnatally (CPS + SD). Fold change in CPS + SD offspring is shown in red, and that of LD + HFD is in black. Data are expressed as mean of fold changes and were analyzed using Ingenuity Pathways Analysis (IPA).

In contrast, comparison of WAT from CPS fed with SD and LD fed with HFD *vs.* both LD fed with SD show striking similarity and only four proteins were differentially expressed in CPS HFD. Taken together, these proteomics results support the notion that in terms of protein expression, WAT from adult CPS offspring is somewhat similar to WAT from adult LD offspring fed with HFD. This possibility was further investigated by running functional proteomic analyses to target common protein profiles by these means; 20 differentially expressed proteins were identified in both experimental groups as compared to the adult offspring that had been gestated under control LD conditions receiving SD postnatally (**Figure 9**). Of note, a most important difference in WAT was found in MYADM, that was −9.7 times downregulated regarding LD + HFD. So far, the actual role of MYADM in adipose tissue function remains non-investigated, but recent evidence is in line with a potential role of this protein in local inflammation, as mediator of low-grade inflammation as deduced for its role in membrane (34). After this exploratory analysis, we decided to focus on the common proteins for the CPS and LD + HFD groups, based on our observed results. Under this criterion, we selected the 20 proteins shown in **Figure 9**, grouped to build a new dataset and analyzed by IPA to generate the interactomes.

**Figure 10:**
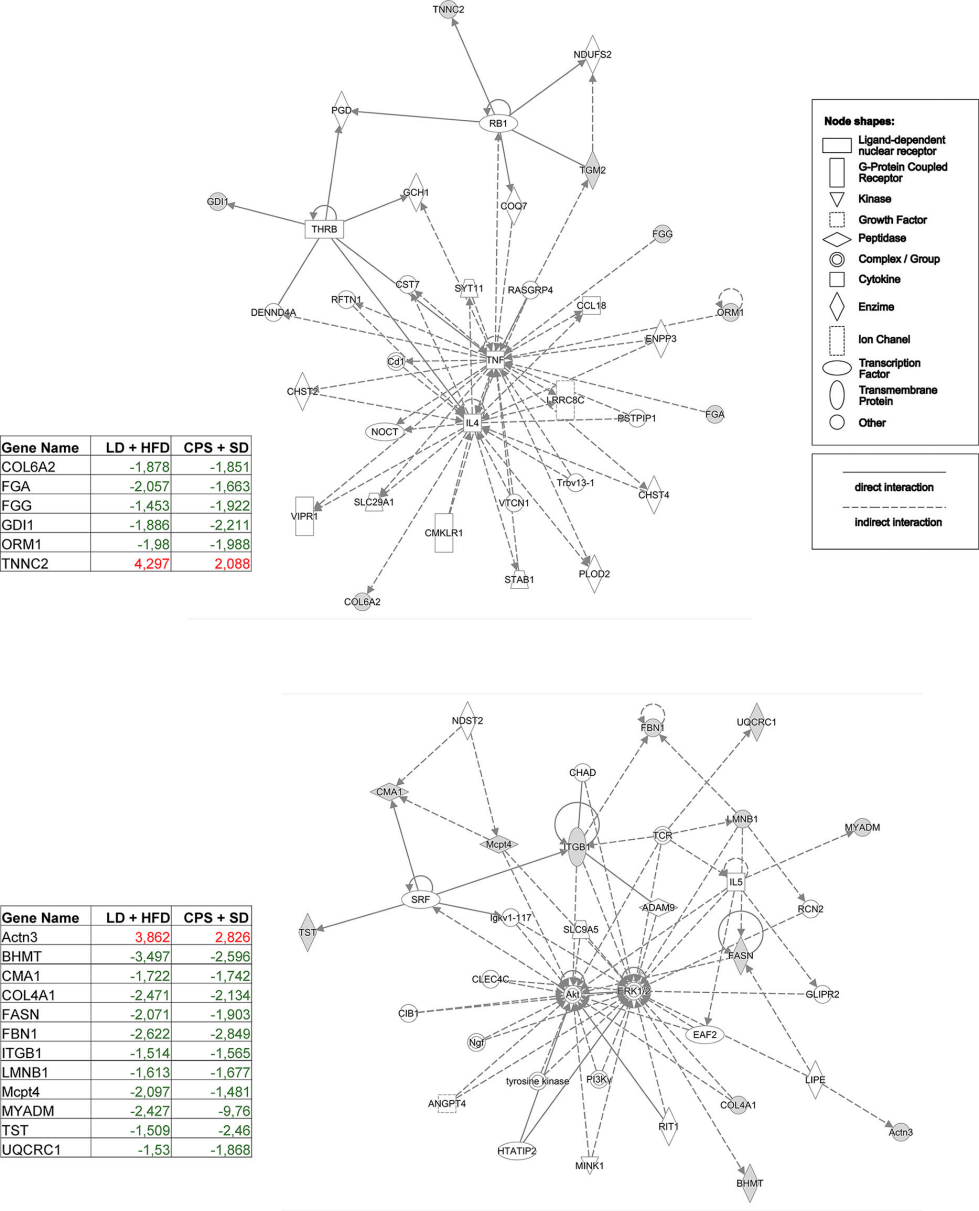
Interactome depicting the pathways that were most significantly enriched by differentially expressed proteins identified by quantitative proteomics of white adipose tissue (WAT) from adult offspring. Comparison: adult offspring that had been gestated under control (LD) conditions and fed with high-fat diet (HFD) during 12 weeks from postnatal day 100 onwards (LD + HFD group) *versus* adult offspring that had been gestated under CPS conditions and fed with standard diet postnatally (CPS + SD). Red, upregulated protein; green, downregulated protein; white, protein related in the network without expression changes. Proteins connected with solid lines have direct links; those connected with dotted lines have indirect links. The set of proteins was grouped in the Ingenuity Top Network 1: Cellular Development, Cell Morphology, Cell-Mediated Immune Response, and Inflammatory Response pathways.

Detailed pathway analysis of WAT from adult CPS + SD showed an imbalance in the pathways related to inflammatory status, network 1 (TNF/IL4, score 14; **Figure 10**, top panel), and network 2 (AKT/ERK, score 28; **Figure 10**, lower panel) supporting the idea that WAT from adult CPS offspring fed with SD was undoubtedly affected to an important extent by gestational chronodisruption.

## Discussion

Life on Earth has evolved over million years to successfully adapt to dominant environmental clues such as light/dark cycles. However, several environmental factors have been influenced by human activity, with artificial light at night (ALAN) being one of the most recent changes imposed over roughly the last century. ALAN is linked not only to light pollution of densely populated locations but also to shift work schedules (about 20% of the global workforce). Recent research on the crosstalk between metabolism and the circadian system has provided evidence linking shift work schedules with the onset of metabolic disorders such as obesity, type 2 diabetes, and cardiovascular dysfunction (35). Considering that a clear relationship has been established for an adverse intrauterine milieu with chronic disease later in life [references in (36, 37)], we tested the hypothesis that gestational chronodisruption (CPS conditions) translates into long-term detrimental effects on glucose homeostasis and adipose tissue physiology, increasing the negative impact of HFD as a cardiometabolic challenge during adulthood in the male offspring.

Gestation under a CPS has an important effect on the adrenal and cardiovascular system in the male offspring during adulthood (6, 7, 30). In the present paper, we found the impact of CPS on adipose tissue recruitment and function under standard diet that were enhanced by the HFD challenge. At 100 days of age, several differences were present between the LD and CPS group. We observed significant differences in intraperitoneal glucose tolerance test, requiring more time to return to the basal levels, accompanied by an increase in leptin, suggesting an increase of adipose tissue that may be involved in the early metabolic effect described above. In addition, animals gestated under CPS conditions responded to HFD with altered iGTT and iITT profiles, in parallel with increased weight gain without any change in food intake. In keeping with early CPS effects on adipose tissue, at 100 days of age in WAT perirenal, global methylation displays differences between LD and CPS, with CPS offspring showing lower levels of global genomic DNA methylation during daytime. Altogether, the current results, before the challenge using HFD, support the idea that an epigenetic mechanism should be present in the early effect observed in WAT at 100 days of age. Next, we found that chronodisruption throughout gestation induced significant changes in body weight and adipose tissue recruitment and function under standard diet. CPS effects on adipose tissue were exacerbated by HFD. By 200 days of age and after 12 weeks of receiving HFD, the distribution of different adipose tissue depots was altered. Besides, the amount of inguinal white adipose tissue (iWAT) was increased in the adult CPS offspring, while the amount of interscapular brown adipose tissue (iBAT) decreased significantly. Furthermore, the *in vitro* lipolytic capacity of adipose tissue from CPS offspring (evaluated by glycerol production) was inhibited in animals exposed to prenatal CPS and postnatal HFD. At 200 days old (after 12 weeks with HFD), an obesogenic phenotype was observed in CPS animals, which, at the molecular level, might be explained by the altered expression pattern found for 275 proteins in CPS adipose tissue, as compared with control (LD) animals fed with a standard diet. Interestingly, the CPS + SD and LD + HFD groups showed 20 differentially expressed proteins in common (2 upregulated and 18 downregulated). Based on these common proteins, functional analyses identified two functional pathways as significantly altered: network 1-TNF/IL4 and network 2-AKT/ERK. Altogether, the present results support the notion that white adipose tissue function was programmed *in utero* by chronodisruption, inducing changes in physiology and metabolic response capacity that persist into adulthood. Interestingly, the key results in the current manuscript support the idea that CPS offspring treated with a standard diet, in terms of metabolic assessment, are close to a control animal fed with a HFD.

Increased accumulation of white adipose tissue has been proposed as a strong and independent predictor of adverse health outcomes associated with obesity (38), as reported here. Besides its role in energy storage, WAT is also an active secretory organ producing a large number of molecules termed adipokines. Actually, adipokines participate in the modulation of glucose and lipid homeostasis *via* the central effects of leptin (39). Adipokines include pro-inflammatory factors and chemokines, increased in obesity (40). Although obesity has been associated with increased accumulation of macrophages within the fat mass, it remains unclear how the crosstalk between macrophages and white adipocyte tissue initiates the dysfunction of white adipose tissue in metabolic disorders. Recently, it has been demonstrated that a link between metabolic syndrome and chronodisruption is provided by the early onset of low-grade inflammation (38, 41), an inflammatory state associated with a wide range of chronic conditions, such as metabolic syndrome, type 2 diabetes mellitus, and cardiovascular disease (42). Reinforcing this issue, our current results plus other works of our group show that maternal chronodisruption is translated in the offspring in an obesogenic phenotype in which low-grade inflammation is already present at 90 days of age (30); it must be kept in mind that these findings are consistent with the significant range of differentially expressed proteins identified by means of quantitative proteomics in WAT.

We suggest that chronodisruption interfered with the normal developmental trajectory of adipose tissue *in utero*, which led to readily consequences for metabolism during adult life. The persistent deleterious effects secondary to light at night along gestation suggest the involvement of epigenetic mechanisms. Indeed, a significant change in the levels of global genomic DNA methylation between day and night was detected in white adipose tissue. On the other hand, *in vitro* glycerol production in response to norepinephrine was affected. Therefore, regulatory mechanisms related to the misalignment of circadian rhythms may operate in WAT. In this context, it is worth mentioning that in a recent publication, Moreno-Mendez et al. (43) argued that epigenetic impairment of adipose tissue function might derive from a mechanism involving hypomethylation of IGF2 and hypermethylation of leptin and TNF-A. This is consistent with our findings that independent of the diet, animals exposed to chronodisruption during gestation between 100 and 200 days of age are metabolically comparable to those LD animals fed with HFD. A series of cases reported by Nahme et al. (44) in 2019 suggest that these findings can be extrapolated to human health. These authors, for the first time, observed a direct relationship between misalignment of the melatonin rhythm during pregnancy and gestational problems together with a low Apgar score in the newborns from these mothers. While we must be careful to overinterpret a series of cases, there are already many studies suggesting an increased risk of adverse pregnancy outcomes secondary to maternal shift work throughout pregnancy (12) and in their offspring (45, 46). For instance, it has been demonstrated that deficient nutrition during gestation alters the development of adipocytes *in utero*, resulting in a permanent increase in the ability to form new adipocytes in adipose tissue depots and increase lipid storage in existing adipocytes (47).

In addition, we know that maternal melatonin during pregnancy may also play an important role in the development of brown adipose tissue and the thermoregulation of the newborn sheep (48, 49). Circadian disruption chronically impairs the biological clock’s function, favoring multiple pathological processes like cancer and metabolic and cardiovascular disorders (50). Many of these processes could be related to a low-grade inflammation state, as suggested by the upregulation of TNF-a, IL-1b, and IL-6 in various tissues from rat and other animal models of chronodisruption in sleep deprivation (51, 52). Of note, our proteomic results suggest changes in the same direction. Low-grade inflammation has been implicated in the development of chronic diseases. For instance, undernutrition *in utero* causes impairment in muscle growth during fetal growth, and after birth, these individuals accumulate a disproportionately high-fat mass (53). The adipose tissue secretes several potent inflammatory factors, which may lead to low-grade inflammation. Though there are scarce evidence that links gestational chronodisruption with a low-grade inflammation state, our results suggest that gestational chronodisruption promotes an increase of pro-inflammatory cytokines in males (30), which could be the central mechanism of programming observed here. Consistent with this evidence, we observed that two pivotal pathways involved in adipose tissue function like TNF/IL4 and AKT/ERK were modified in LD fed with HFD and CPS with standard diet at 200 days of age; both pathways involved a key step in the progression of metabolic adipose tissue dysfunction like low-grade inflammation and differentiation process (54). Therefore, we speculate that part of this key pathways was modified *in utero* and could be one of the mechanisms programming *in utero* by gestational chronodisruption, an idea that needs more exploration. In the same line, results of our group support the notion that a critical organ involved in metabolism and correct response to stress, the adrenal gland, is programmed *in utero* by maternal chronodisruption, in which CPS offspring present a desynchronization of the adrenal circadian clock and steroidogenic pathway, leading to abnormal stress responses and potentially increasing the risk of developing chronic diseases (6, 7).

An interesting comparison of our results with those available in sheep suggests to us that sheep are more resilient than the rat to the impact of photoperiod changes during gestation (21, 55). The evidence supports those minor changes that occur in pregnant sheep in comparison to the pregnant rat as well as the minor changes observed in the young lamb offspring, opening the possibility to speculate that a protective physiological environment allows the sheep to cope with adverse environmental cues. How this occurs remains to be investigated, but we need to keep in mind that the sheep present several physiological differences compared to rats, such as glucocorticoid production, which is low in sheep (56), pregnancy physiology, and the seasonal characteristic of the sheep and postnatal development. Further research is needed in order to establish how both animal models help to understand the long-term impact of gestational chronodisruption in humans.

In summary, the present results in male rats strongly support the idea that gestational chronodisruption may act through epigenetic mechanisms to define an abnormal adipose tissue phenotype, which is functionally consistent with increased risk of obesity, insulin resistance, hypertension, and metabolic syndrome. This liability seems to be compounded by HFD, which can be considered a highly prevalent risk factor in modern life.

## Data Availability Statement

The raw data supporting the conclusions of this article will be made available by the authors, without undue reservation. Protein data are available via ProteomeXchange with identifier PXD026315 https://www.ebi.ac.uk/pride/archive/projects/PXD026315.

## Ethics Statement

The animal study was reviewed and approved by Bioethics Commission from the Universidad Austral de Chile (CBA: 352/2019).

## Author Contributions

DH, CT-F, and HR conceived and designed the study, analyzed and interpreted the data, drafted the manuscript, critically revised important intellectual content in the manuscript, and provided overall supervision. NM, TK, CS, ES, KV, FT, and MS-F performed the experiments, analyzed the data, drafted the manuscript, and contributed to intellectual content in the manuscript. All authors contributed to the article and approved the submitted version.

## Funding

This work was supported by Grants Fondecyt 11190711 (DH), Fondecyt 1191207 (CT-F), and Fondecyt 11170245 (NM) from Fondo Nacional de Desarrollo Científico y Tecnológico, Chile.

## Conflict of Interest

The authors declare that the research was conducted in the absence of any commercial or financial relationships that could be construed as a potential conflict of interest.

## Publisher’s Note

All claims expressed in this article are solely those of the authors and do not necessarily represent those of their affiliated organizations, or those of the publisher, the editors and the reviewers. Any product that may be evaluated in this article, or claim that may be made by its manufacturer, is not guaranteed or endorsed by the publisher.

## Supplementary Material

The Supplementary Material for this article can be found online at: https://www.frontiersin.org/articles/10.3389/fendo.2021.678468/full#supplementary-material
