# Supplementary figures and images for: Maternal Chronodisruption Throughout Pregnancy Impairs Glucose Homeostasis and Adipose Tissue Physiology in the Male Rat Offspring
Source: Front Endocrinol (Lausanne). 2021 Aug 16;12:678468. doi: 10.3389/fendo.2021.678468 (PMC8415792; doi:10.3389/fendo.2021.678468)

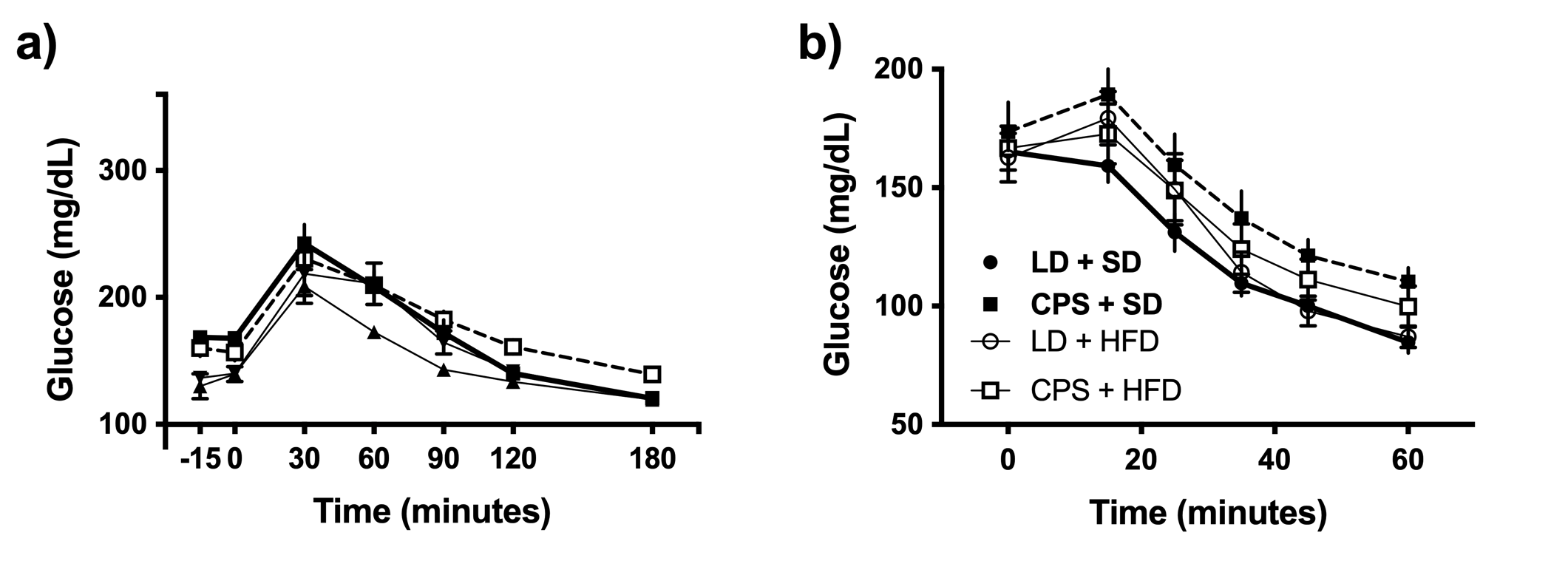

Supplement: Supplementary file 1 [file Image_1.tiff]
